# Studying Human Fertility and Environmental Exposures

**DOI:** 10.1289/ehp.112-1247502

**Published:** 2004-08

**Authors:** Rémy Slama, Béatrice Ducot, Niels Keiding, Jean Bouyer

**Affiliations:** INSERM and INED (French National Institute for Health and Medical Research and the French National Institute for Demographic Studies), Unit 569, Le Kremlin-Bicêtre, France, E-mail: slama@vjf.inserm.fr; Department of Biostatistics, University of Copenhagen, Copenhagen, Denmark; INSERM and INED (French National Institute for Health and Medical Research and the French National Institute for Demographic Studies), Unit 569, Le Kremlin-Bicêtre, France

In their review of approaches to studying the influence of environmental exposures on human fecundity, Tingen et al. (2004) compared several ways of assessing fecundity.

Fecundity—the probability of pregnancy in couples having regular intercourse without contraception—can be assessed by applying appropriate statistical approaches to time-to-pregnancy (TTP) data. Tingen et al. (2004) provided a thorough presentation of the detailed prospective approach to assess TTP. We agree that advantages of this approach, in which daily urine samples are collected, include allowing the estimation of the daily probability of pregnancy within a menstrual cycle and studying the early survival of the embryo; however, we have reservations about the authors’ conclusion that the detailed prospective approach should be seen as the gold standard for studying the effects of environmental exposures on fecundity.

We believe that prospective TTP studies, whether detailed or not, have one main limitation, which lies in the difficulty of defining precisely the target population: These studies are often based on the inclusion of couples soon planning to attempt conception or to stop using contraceptive methods. In our opinion, this population is ill-defined and lacks a sampling frame, which makes the estimation of participation rates difficult. Indeed, many published detailed prospective TTP studies had unreported or low participation rates (Buck et al. 2004), opening the door for selection biases. We also doubt that these “super pregnancy planners,” who program their pregnancy attempts months ahead, are representative of the general population. For example, detailed prospective TTP studies have sometimes included couples with higher-than-average educational level (Wilcox et al. 1988) or those who use natural family planning methods not widely used (Dunson et al. 2002). These characteristics may be associated with the probability of pregnancy and with the environmental exposures of interest, thus resulting in possible biases.

These limitations of the prospective approach do not justify a preference for retrospective studies. As pointed out by Tingen et al. (2004), the exclusion of infertile couples in most retrospective studies is indeed of particular concern; it reduces statistical power and leads to underestimation of the effect of the environmental exposure of interest (Slama et al. 2004).

The current duration approach, another approach not mentioned by Tingen et al. (2004), makes it possible to include infertile couples without resorting to detailed prospective studies. The current duration approach relies on the inclusion of couples currently trying to conceive or who are having intercourse without contraception (Keiding et al. 2002; Olsen and Andersen 1999). The recruited couples are asked how long they have been having unprotected sexual intercourse. Follow-up of these couples is not required (Keiding et al. 2002), but it is possible to obtain information on the occurrence of a pregnancy. In this case, the approach is based on principles from the case–cohort design (Olsen and Andersen 1999).

In the current duration approach, data on the frequency of sexual intercourse, the duration of the menstrual cycle during the attempt at pregnancy, and environmental exposures can be collected with virtually no recall bias. The collection of urine or other biologic samples is possible, at least from the date of inclusion; that is, some time after cessation of contraceptive use. The advantage of the current duration approach is that the inclusion criterion (currently having sexual intercourse without contraception) is more clear-cut than that of the prospective approach. This approach thus has a clearly defined sampling frame. We are currently testing this approach on a representative population of French women 18–45 years of age.

The four approaches to assessing TTP are based on different inclusion schemes. The retrospective approach is based on the inclusion of couples who already had a pregnancy; prospective approaches (detailed and not) are most often based on the inclusion of couples who will soon discontinue contraceptive use; and the current duration approach is based on the inclusion of couples currently trying to conceive. We believe that none of these methods can currently be considered a gold standard. In particular, unlike Tingen et al. (2004), we do not think that the potential bias from the exclusion of pregnancies occurring during contraceptive use (Baird et al. 1994) is specific to the retrospective approach, because prospective (and current duration) studies seldom include couples using contraceptive methods.

Instead, we believe that the existence of new, alternative approaches should provoke comparative studies, leaving room for debate before conclusions are drawn about which approach is preferable for a given purpose.
